# Consciousness, Cognition and the Neuronal Cytoskeleton – A New Paradigm Needed in Neuroscience

**DOI:** 10.3389/fnmol.2022.869935

**Published:** 2022-06-16

**Authors:** Stuart Hameroff

**Affiliations:** ^1^Department of Anesthesiology, The University of Arizona, Tucson, AZ, United States; ^2^Department of Psychology, The University of Arizona, Tucson, AZ, United States; ^3^Center for Consciousness Studies, The University of Arizona, Tucson, AZ, United States

**Keywords:** microtubules, tubulin, consciousness, Orch OR, quantum coherence, cortical pyramidal neurons, quantum computing, memory

## Abstract

Viewing the brain as a complex computer of simple neurons cannot account for consciousness nor essential features of cognition. Single cell organisms with no synapses perform purposeful intelligent functions using their cytoskeletal microtubules. A new paradigm is needed to view the brain as a scale-invariant hierarchy extending both upward from the level of neurons to larger and larger neuronal networks, but also downward, inward, to deeper, faster quantum and classical processes in cytoskeletal microtubules inside neurons. Evidence shows self-similar patterns of conductive resonances repeating in terahertz, gigahertz, megahertz, kilohertz and hertz frequency ranges in microtubules. These conductive resonances apparently originate in terahertz quantum dipole oscillations and optical interactions among pi electron resonance clouds of aromatic amino acid rings of tryptophan, phenylalanine and tyrosine within each tubulin, the component subunit of microtubules, and the brain’s most abundant protein. Evidence from cultured neuronal networks also now shows that gigahertz and megahertz oscillations in dendritic-somatic microtubules regulate specific firings of distal axonal branches, *causally modulating* membrane and synaptic activities. The brain should be viewed as a scale-invariant hierarchy, with quantum and classical processes critical to consciousness and cognition originating in microtubules inside neurons.

## Overview – The Need for a New Paradigm in Neuroscience

Since the mid-20th century, the brain has been viewed as a complex computer of simple neurons, each acting as a fundamental algorithmic unit, e.g., Hodgkin-Huxley “integrate-and-fire” threshold logic device (Hodgkin and Huxley, 1952). Within these units, neuronal-level information relevant to cognition and consciousness is presumed to be mediated entirely by neuronal outer surfaces, e.g., membrane receptors and ion channels, dendritic-somatic integration potentials, axonal firings and synaptic transmissions. Strengths of synaptic connections are presumed to regulate flow in membranes and synapses of electrochemical information through computational neuronal networks. Cognition and consciousness emerge at critically complex “higher order” levels of computation among relatively simple fundamental neuronal units. But these “higher order” levels are not well defined, and neurons are not really that simple. The brain-as-computer approach can’t account for critical aspects of cognition (see below), nor the “hard problem” of conscious experience (Chalmers, 1996).

Are neurons really that simple? Single cell organisms like amoeboid *physarum* (Adamatzky and Jones, 2010) and the nimble *paramecium* can solve complex problems, move and swim to find food and mates, learn and have sex, all without synaptic connections. Whether conscious or not, they perform these cognitive functions through purposeful activities of their internal cytoskeleton, including microtubules, and microtubule-based cilia. All animal cells have a cytoskeleton, and asymmetrical neurons have a particularly prominent, active and uniquely arrayed cytoskeleton including microtubules, self-assembling cylindrical polymers of the protein tubulin. Microtubules’ hexagonal grid-like lattice geometry and purposeful behaviors have prompted the idea that microtubules process information. They appear capable of deeper, faster activities related to cognitive functions *within* neurons, including modulation of axonal firings (Singh et al., 2021). The “Hodgkin-Huxley” membrane-only neuron may be an insult to actual neurons.

Is consciousness really a computation? In his book *The emperor’s new mind*, Sir Roger Penrose (1989) suggested that consciousness was not entirely algorithmic, that it required a “non-computable” factor outside the brain’s intrinsic neuronal computation. The only possible non-computable source, he further contended, was outside classical physics, a proposed self-collapse, or “objective reduction” (“OR”) of quantum superposition states—a “collapse of the quantum wavefunction.” But quantum state reduction was, and is, yet another mystery. In the early 20th century, it appeared to scientists including Niels Bohr that quantum particles could exist in superposition of multiple co-existing locations or states. But when measured, or consciously observed, superpositions would apparently “collapse,” or reduce to particular states and locations. Quantum physicists John von Neumann, Eugene Wigner, and more recently Henry Stapp and philosopher David Chalmers have taken the view that conscious observation *causes* quantum state reduction, that consciousness “collapses the wavefunction” (Chalmers and McQueen, 2022). However, this view is “dualist,” leaving consciousness and quantum superposition unexplained and outside science.

Rather than consciousness causing collapse, Roger Penrose took an alternate view, proposing that collapse occurred naturally, and that collapse caused consciousness. He suggested that quantum state reduction—collapse of the wavefunction—is due to an objective threshold (“objective reduction,” “OR”) in the fine scale structure of the universe, that such events *produced* the rudiments of phenomenal conscious experience (or “qualia”).

To do so, and based on Einstein’s general relativity, Penrose explained superpositions as tiny separated curvatures in spacetime geometry. Were such separations to continue, one might imagine, each spacetime curvature would evolve its own universe, as suggested in the “Many Worlds” hypothesis (Everett, 1957). However, Penrose deduced that spacetime separations would be unstable, and undergo OR based on the quantum uncertainty principle at time *t* = *ħ/E_G_* (*ħ* is the Planck-Dirac constant, and *E*_*G*_ the gravitational self-energy of the superposition—(Penrose, 1989, 1996; Hameroff and Penrose, 1996a).

When this OR threshold is met, according to Penrose, specific spacetime curvatures and states of classical reality are abruptly selected, and a quantum-like unit of phenomenal experience, a “quale” (plural “qualia”) occurs. Rather than consciousness causing collapse, collapse causes consciousness (or is equivalent to it). Particular states and qualia selected in each OR event would be chosen neither randomly, nor algorithmically, but influenced “non-computably” by “Platonic values” intrinsic to the universe (Moroz et al., 1998).

In a thermal microenvironment, such OR events would be ubiquitous, and their qualia random, disconnected and thus merely “proto-conscious” (metaphorically like the random notes and tones of an orchestra “tuning up”). How could proto-conscious OR events be “orchestrated” in the brain for full conscious experience and causal action?

In the mid 1990s, Penrose and Hameroff suggested that microtubules inside brain neurons *orchestrate* quantum vibrational superpositions through resonance, entanglement and memory, guiding wavefunction evolution to threshold for “orchestrated OR” (“Orch OR”), events proposed to result in moments of full, rich conscious experience (more like music than random notes and tones). Sequences of such moments would give rise to our “stream of consciousness” (Penrose and Hameroff, 1995; Hameroff and Penrose, 1996a,b, 2014a).

Orch OR and quantum biology in general were viewed skeptically, largely because technological quantum computers must operate near absolute zero temperature to avoid disruption by random, thermal “decoherence” in the microenvironment. In biology the microenvironment is usually assumed to be a polar, aqueous medium. Living cells are 70 percent water, which at biological temperatures at the microscale would be chaotic and disruptive to quantum coherence. However, cells are highly heterogeneous, and include regions inside lipids, proteins, nucleic acids and other biomolecules which have non-polar inner regions, and support quantum coherence rather than disrupt it. Moreover, at least some intra-cellular water may be ordered by coupling with periodic, coherently oscillating charges on outer surfaces and inner cores of microtubules.

One way to correlate localized regions with their function is through pharmacology, i.e., solubility and binding “compartments” where drugs bind and act to exert their specific effects. Most drugs are polar, and soluble in similarly polar blood, water and biological fluids to easily reach receptors on cell outer membrane surfaces.

On the other hand, anesthetic gases are decidedly non-polar, and selectively block consciousness, sparing non-conscious brain activity. Their site and mechanism of action may point to the origin of consciousness. Non-polar, lipid-like, or “oil-like,” anesthetics are poorly soluble in polar, aqueous media (“oil and water don’t mix”). And strangely, anesthetics gases are basically inert, and don’t form chemical bonds with their target sites. They attract and weakly bind by quantum-level, van der Waals forces (see below).

Inhaled along with oxygen into the lungs, the non-polar anesthetic molecules quickly transit through blood and fluids to reach non-polar, oil-like regions inside molecules within tissues and cells all over the body. There they bind in large amounts by weak, quantum-level van der Waals forces, specifically “London dipole dispersion forces.” And yet anesthetics have few effects on bodily functions other than selectively blocking consciousness. Could consciousness—and only consciousness—involve highly “orchestrated,” thus easily perturbable, quantum processes? If so, where might they be?

Despite having near-identical effects in selectively blocking consciousness, anesthetic gases have different types of chemical structures, e.g., ethers, halogenated hydrocarbons, nitrous oxide and the inert element xenon. They have in common their “non-polarity,” a determinant of solubility.

At the turn of the 20th century, Hans Meyer (1899) and Charles Ernst Overton (1901) tested a group of gases for anesthetic action, cataloged their respective potencies in rendering animals unresponsive and apparently unconscious, and then sought a physical parameter which might correlate with anesthetic potency. Working independently, Meyer and Overton concluded that, over many orders of magnitude, with a dozen or more gases, there was a strong correlation between anesthetic potency and solubility in non-polar, oil-like “hydrophobic” (water-aversive) regions closely resembling benzene and olive oil, a relationship which became known as the “Meyer-Overton correlation.” The particular anesthetic-soluble regions were later characterized by a low Hildebrand solubility coefficient lambda (15.2–19.3 SI Units), again closely resembling benzene and olive oil (Hameroff, 2006).

Where in the brain, in *which* particular intra-molecular non-polar regions, do anesthetics act to block consciousness? Initially, lipid regions of membranes were considered, then various protein receptors and/or ion channels embedded in neuronal membranes were assumed to mediate anesthetic action, e.g., post-synaptic receptor. But over decades, extensive research failed to find any correlation between anesthetic potency and membrane receptor effects (Eger et al., 2008). Anesthetics also bind in non-polar regions in tubulin in microtubules, and genomic, proteomic and optogenetic research pointed to tubulin and microtubules as the target of anesthetic action (Xi et al., 2004; Pan et al., 2007; Emerson et al., 2013).

Non-polar anesthetic binding sites in tubulin (and other proteins) are comprised largely of “pi” electron resonance clouds of benzene-like organic rings in aromatic amino acids tryptophan, phenylalanine and tyrosine. The basis for organic chemistry, “pi” electron resonance clouds are delocalized quantum objects, non-polar, but polarizable, and can induce and couple quantum dipoles, participate in quantum optical effects, and oscillate coherently in terahertz frequency ranges (Lundholm et al., 2015).

**FIGURE 1:**
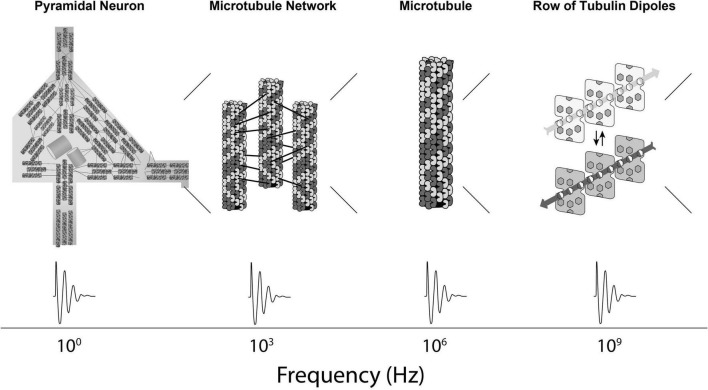
First part of a proposed spatiotemporal hierarchy in which Orch OR-mediated consciousness can occur, starting here inward from the level of neurons: From left to right, a cortical pyramidal neuron showing internal networks of mixed polarity microtubules, a network of mixed polarity microtubules, a single microtubule, a row of tubulins within a microtubule with schematic display of collective dipoles among pi electron resonance rings. Below—frequency dynamics at relevant scales.

**FIGURE 2:**
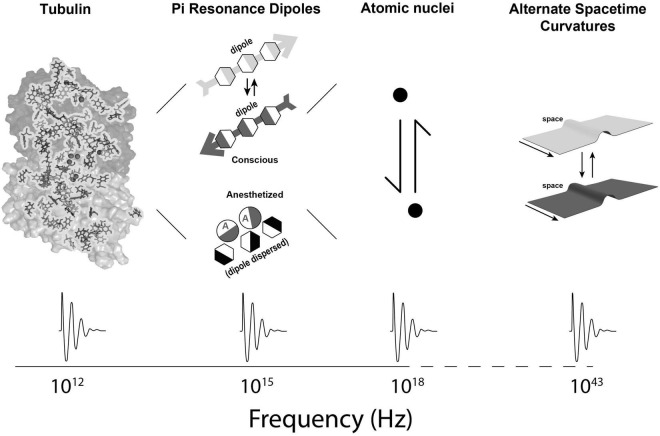
Second part of a spatiotemporal hierarchy in which Orch OR-mediated consciousness can occur: From left to right, a single tubulin protein showing internal pi resonance amino acids, pi resonance dipoles with effect of anesthetic gases dispersing dipoles, atomic nuclei, and (much smaller) curvatures in fundamental spacetime geometry. At bottom, dynamical patterns of activity are shown at different spatiotemporal levels (hierarchical frequencies taken from Craddock et al., 2012a; Sahu et al., 2013a,b, 2014; Saxena et al., 2020).

In Orch OR, terahertz quantum vibrations originate among pi electron resonance clouds in tubulins, extending to neighboring tubulins in helical pathways reaching mesoscopic and macroscopic scales—the “quantum underground” (Craddock et al., 2016). Terahertz quantum vibrations are proposed to resonate and interfere in a fractal-like hierarchy with self-similar dynamics spanning gigahertz, megahertz, kilohertz and hertz frequencies, across progressively larger, slower scales into the range of EEG and cognitive events (Figures 1, 2). This scale-invariant hierarchy would enable Orch OR to operate and occur at times t = ħ/E_G_, across multiple scales, providing a spectrum of repetitive conscious moments, and stream of consciousness.

Irrespective of Orch OR, information processing relevant to cognitive and conscious brain functions seem likely to extend inward in faster, smaller scale dynamics to cytoskeletal microtubules. With a lack of progress through brain-as-computer approaches, a new paradigm in neuroscience is needed.

There is evidence for a microtubule-based, scale-invariant hierarchical system (Sahu et al., 2013a,b; Sahu et al., 2014; Saxena et al., 2020). When alternating current (“AC”) electrical energy is applied to tubulin and/or microtubules, there is generally poor conductance—tubulin and microtubules are good insulators. However, at certain, specific “resonant” applied AC frequencies, conductance is extremely high and microtubules are excellent (“ballistic”) conductors. These resonant frequencies show self-similar conductance patterns repeating every 3 or so orders of magnitude, over 15 orders of magnitude in the brain, from the quantum world to the electro-encephalogram (“EEG”). From terahertz through gigahertz, megahertz, kilohertz and hertz, conductance at each of these frequencies showed self-similar “triplet-of-triplet” resonance patterns.

Other work indicates microtubules have endogenous oscillations in these same frequencies, which can regulate slower membrane and synaptic activities (e.g., Singh et al., 2021). Cantero et al. (2016) (c.f. Cantero and Cantiello, 2020) have shown direct oscillations from microtubules and tubulin sheets in the range of 100 Hz, “high gamma” EEG. Microtubules may also host quantum optical states such as sub-radiance and super-radiance (Celardo et al., 2018).

How do systems communicate across scale? Scale-invariance described by “1/f” power laws are observed in many natural fractal-like phenomena, from field theories to fluid dynamics to cosmology. In neuroscience, the electro-encephalogram (“EEG”) shows self-similar patterns, or nested frequencies over several orders of magnitude and hundreds of hertz, presumably based on individual neurons, and larger, slower networks of neurons (He et al., 2010). The new paradigm described here also extends inward, inside neurons, totaling over 15 orders of magnitude in the brain (Figures 1, 2), and has significant explanatory power (see further below). The critical features required for the new paradigm are (1) classical and/or quantum information processing in microtubules inside brain neurons, and (2) capabilities for both top-down, and bottom-up regulation to and from microtubules in a scale-invariant brain hierarchy. These features do appear feasible.

## Information Processing in Microtubules and the Cytoskeleton

The first reference to cytoskeletal information processing appears to have come from famed neuroscientist Charles Sherrington (1951). Pondering the intelligent behavior and internal structure of single cell organisms, he surmised “of nerve there is no trace, but perhaps the cyto-skeleton might serve.”

**FIGURE 3:**
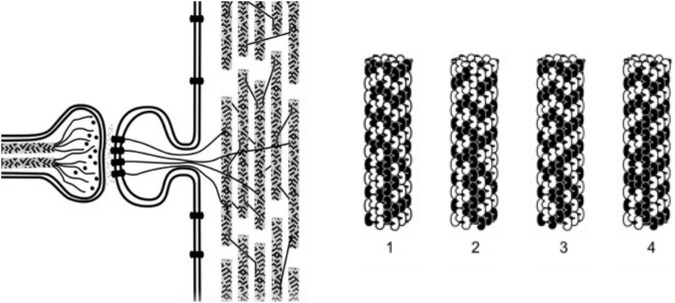
**Left:** An axon forms a synapse with a. dendritic spine on a dendrite of another neuron, internal microtubules, actin and membrane proteins are shown. Dendritic microtubules are interrupted, and in mixed polarity arrays. **Right:** 4 steps in a microtubule automata processing information with binary states of tubulins (microtubule automata patterns on right taken from Rasmussen et al., 1990).

But how? When the structure of cytoskeletal microtubules was recognized through X-ray crystallography to be a cylindrical lattice with Fibonacci geometry (Amos and Klug, 1974), an idea emerged that microtubules’ purposeful actions could possibly result from computer-like information processing. For example discrete states of individual tubulin subunit proteins could represent and exchange information within microtubule lattices. The state of each tubulin would correspond with binary “bit” states in computers and automata (Figure 3). For example, Hameroff and Watt (1982) suggested microtubules acted as Boolean switching matrices, processing binary bit states moving through microtubules and microtubule-associated proteins (c.f. Hameroff, 1987). Simulated networks of 2 “microtubule automata,” connected by microtubule-associated proteins through which bit states could also propagate, were found to show unsupervised learning (Rasmussen et al., 1990).

Automata and computer systems generally utilize some type of periodic clocking mechanism to synchronize and update operations. In the microtubule automata simulations done by Rasmussen et al. (1990), collective coherent dipole oscillations among tubulins provided coherent time steps for synchronized updates, for example steps 1–4 in Figure 3 on the right. The oscillation updates result in propagating patterns of information, the biological mechanism for this clocking ascribed to “Fröhlich coherence.”

**FIGURE 4:**
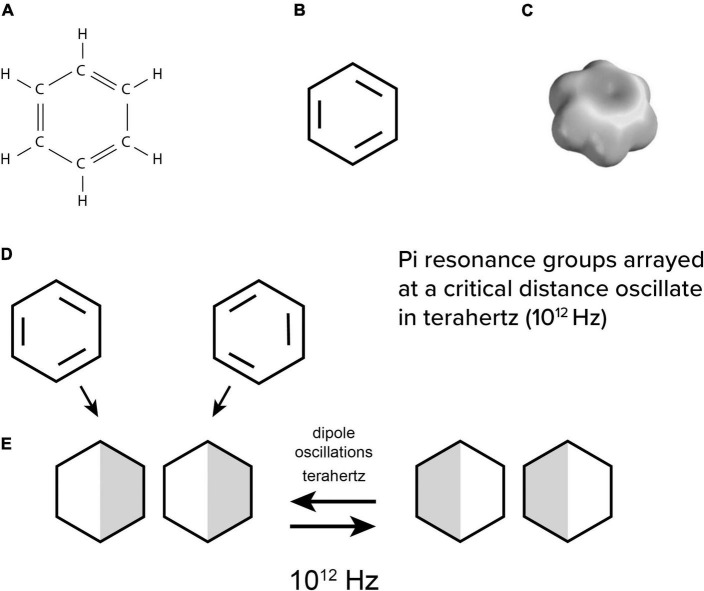
**(A)** chemical structure of benzene, **(B)** simplified with 3 extra electron bonds, and **(C)** with a delocalized electron cloud enveloping the ring. **(D)** individual benzene rings attract by induced dipoles which then **(E)** oscillate in terahertz.

Biophysicist Herbert Fröhlich (1968, 1970, 1975) suggested that dipoles in non-polar intra-protein regions would oscillate due to the most basic structure in organic chemistry—the “pi electron resonance” benzene ring (Figure 4). Each of the hexagonal benzene’s 6 carbons has 4 electron bonds to share with 2 hydrogens and 2 neighbor carbons in a planar ring (Figures 4A,B). This leaves 3 extra “pi orbital” resonance electrons which are portrayed as double bonds resonating between different carbons. In molecular orbital theory, the pi electrons combine and “delocalize” in a space-filling electron cloud enveloping all 6 carbons (Figure 4C). Such clouds are chemically neutral and non-polar, but polarizable, leading to interesting quantum vibrational properties.

The clocking mechanism based on Fröhlich coherence suggested that cytoskeletal information processing inside neurons could increase brain information capacity drastically. Estimates of brain capacity based on complex networks of simple neurons consider 10^11^ neurons per human brain, each having about 10^3^ synapses/neuron. These operate at roughly 10^2^ Hz, giving about 10^16^ operations per second for the entire brain (e.g., Moravec, 1986).

However, neurons and glia each have roughly 10^7^–10^9^ tubulins per cell (10^19^–10^20^ tubulins in a human brain), so with each tubulin oscillating between alternative binary states at a Fröhlich frequency of e.g., 10 megahertz (10^7^ Hz), microtubule information processing may be estimated at up to 10^16^ operations per second *per neuron*, and 10^27^ operations per second for the entire brain. But even that may be a gross underestimate, as these microtubule oscillations may occur in a heterogeneous mosaic of interactive information states.

## Top-Down Effects of Neurons and Neuronal Networks on Microtubules

In a scale-invariant hierarchy, different levels interact causally, both “upward,” and “downward” (Figures 1, 2). “Top-Down” effects acting on the cytoskeleton to modulate its function would come from “higher level,” larger scale, slower dynamics in neuronal membranes and synaptic networks. These influences could be in the form of synaptic and membrane-mediated electromagnetic fields, ionic and chemical fluxes, acid-base pH balance, heat, photons and mechanical vibrations, as well as genetically determined states, “post-genetic” (“post-translational”) modifications, phosphorylation, and binding of microtubule-associated proteins (“MAPs”).

Genetic variability of individual tubulins can lead to heterogeneous mosaics of different tubulin isoforms in a microtubule. For example in rat brain microtubules, tubulins may exist as one of 17 different genetic isoforms, whereas only 11 tubulin isoforms occur in other tissues (Lee et al., 1986).

Some “top-down” effects can occur “post-genetically” (“post-translational modifications”, “PTMs”) to cause ongoing structural or chemical changes at specific tubulins in a microtubule lattice, and act as programmed memory. As suggested by Janke (2014), various types of “top-down” PTMs can bind and alter tubulins at specific locations and patterns, the “tubulin code,” a memory medium in which consciousness can occur (c.f. Hameroff and Watt, 1982; Hameroff, 1987). PTMs include removal or addition of particular amino acids at specific locations in the tubulin peptide chain. Most such PTMs occur on C-termini tubulin “tails” which protrude from the microtubule surface into the charged, polar environment (Janke and Magiera, 2020). There are also PTM sites on the internal microtubule lumen, possibly altering flexibility.

Any specific tubulin or combination of tubulins in a neuronal microtubule lattice may also be phosphorylated in another type of PTM, e.g., by the enzyme calcium-calmodulin kinase 2 (“CaMKII”), involved in memory (see section “Discussion,” further below). With each synaptic event, calcium ions enter the neuron and activate hexagonal CaMKII holoenzymes. 6 kinase domains then extend from each side of the holoenzyme, and can bind and phosphorylate up to 6 tubulins at specific microtubule lattice sites simultaneously (Craddock et al., 2012a,b), consequently imparting a form of memory. Thus tubulin-based information processing in microtubules may involve switching between more than merely binary states, oscillations occurring in a heterogeneous mosaic pattern from “top-down” neurons and networks.

Each tubulin in a microtubule lattice can have one of 17 different genetic states, 5 PTM states, 2 phosphorylation states, etc., so overall 22 or more different possible tubulin states comprising heterogeneous “mosaic” microtubules encoding enormous amounts of information, e.g., ∼10^8^ times 2^22^ possible different tubulin mosaic states occurring at, e.g., 10^7^ times per second, per neuron. What purpose might this serve?

Precise placement of the MAP “tau” at specific microtubule lattice sites in neuronal dendrites serves as “traffic signals” for motor proteins carrying molecular cargo for synaptic plasticity (Dixit et al., 2008). Binding sites for structural MAPs determine the architecture and extent of cytoskeletal networks which define cellular morphology, e.g., synaptic plasticity. In addition tubulin coding as memory would modulate vibrational spectra, behaviors, interference and resonances within the cytoskeleton, and the brain hierarchy.

In addition, such a “tubulin code” may “program” and modulate, by “top-down” effects, vibrational spectra of microtubules in terahertz, gigahertz, megahertz, kilohertz and hertz frequencies, such vibrations being the fundamental origins within the brain of cognition and consciousness.

## “Bottom-Up” Effects of Microtubules on Neuronal and Network Function

As described above, in a scale-invariant hierarchy, different levels interact causally, both “upward,” and “downward” in the hierarchy (Figures 1, 2). There appears to be a need for bottom-up processing, as experiments in neuroscience have increasingly demonstrated that neurons are far more sophisticated than Hodgkin-Huxley threshold logic devices. For example, Gidon et al. (2020) showed that dendrites of human cortical pyramidal neurons perform computations previously thought to require multi-layered networks of neurons. How is that accomplished?

Inside active neurons, electromagnetic oscillations in megahertz and gigahertz frequencies occur, emanating from cytoskeletal filaments including microtubules (Singh et al., 2021). Moreover, the oscillations have been shown to modulate axonal firings to cause deviation from Hodgkin-Huxley behavior, playing a functional role. Singh et al. (2021) inserted arrays of “dielectric resonance” nanoprobes into dendrites, soma, axons and axonal branches of neurons in cultured neuronal networks and detected megahertz and gigahertz oscillations. Sahu et al. (2013a,2014), Saxena et al. (2020) had previously reported conductive resonances in isolated microtubules in these same frequencies, but the new findings showed spontaneous megahertz and gigahertz oscillations from cytoskeletal filaments. Electrophysiological membrane potentials were also recorded from the same neurons.

Some axons separate and branch distally, such that specific branches, but not others, selectively “fire” with a given action potential along the main axon. Singh et al. (2021) found that “firing” in specific axonal branches occurred when gigahertz and megahertz oscillations in those branches correlated with similar oscillations in dendritic-somatic regions, irrespective of membrane potentials. In some cases, axonal branches fired when correlated with oscillations in a different neuron’s dendrites and soma, indicating the megahertz and gigahertz oscillations projected outside the neuron, e.g., as “ephaptic” coupling, or possibly quantum entanglement.

Thus, in neuronal networks, axonal firings were regulated by deeper, faster electromagnetic activity in cytoskeletal circuits, independent of membrane potentials and synaptic transmissions. In a bottom-up fashion, intra-neuronal microtubule activities *causally regulate* higher level, larger scale Hodgkin-Huxley membrane and synaptic behavior in neuronal and network functions.

Spontaneous gigahertz emission has also been detected from *Drosophila*, and is blocked by exposure to the anesthetic gas chloroform (Gaitanidis et al., 2021). Deeper level, faster cytoskeletal dynamics inside brain neurons and other cells may be essential features of living systems, and mediate consciousness.

## Quantum Computing and Orch OR

Quantum physics describes reality at small scales, differing markedly from the reality we observe at macroscopic scales. For example atoms and sub-atomic particles can (1) exist in wave-like “superposition” of multiple possible states and/or locations, (2) be connected non-locally over time and space through “quantum entanglement,” (3) condense to unitary coherent states.

Quantum behaviors are difficult to reconcile with our observed “classical” world. For example, we don’t encounter superpositions in our perceived world, as the very act of measurement, or of conscious observation seems to cause superpositions to reduce to definite states—“collapse of the wavefunction.” And the mechanisms by which quantum particles can be non-locally entangled, or condense to coherent unified entities remain unknown. However, these mysterious quantum properties can, in principle, lend themselves to explanations for features of consciousness.

For example, abrupt transition from unconscious, or subconscious possibilities to consciousness, and causal selection of particular actions and perceptions, may be seen as “quantum state reduction,” or “collapse of the wavefunction” (e.g., Penrose, 1989; Hameroff and Penrose, 2014a). And the unitary nature of cognitive binding, and sense of self may be attributed to quantum coherence, entanglement and condensation. Finally, the “hard problem” of the nature of phenomenal experience (e.g., Chalmers, 1996) may require a connection to an intrinsic feature of the universe, e.g., quantum state reduction (Hameroff and Penrose, 1996b).

But technological quantum devices including quantum computers must operate near absolute zero temperatures to avoid thermal decoherence, and the brain has thus been deemed too “warm, wet and noisy” for organized quantum mechanisms. But aromatic pi electron resonance ring structures, which do support quantum processes, are pervasive in biomolecular systems.

When individual aromatic pi resonance rings are near, (Figures 4D,E) they attract each other by quantum van der Waals forces despite a lack of charge. This occurs because the electronegativity of one cloud repels that in the other, forming a pair of coupled dipoles which then (Figure 4D) attract and (Figure 4E) oscillate in terahertz. Coherent resonances and oscillations discovered in terahertz, gigahertz, megahertz, kilohertz and hertz frequency ranges in tubulin and microtubules (Sahu et al., 2013a,b, 2014; Saxena et al., 2020) may represent Fröhlich coherence, discussed above.

**FIGURE 5:**
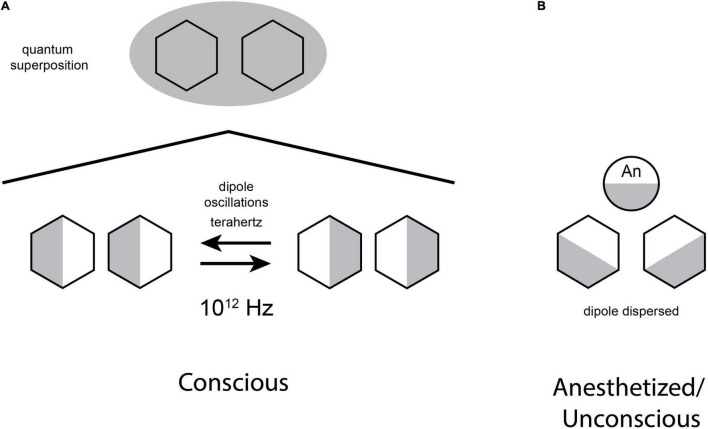
**(A)** Oscillating benzene rings may exist in superposition of both dipole orientations, thus forming a potential quantum bit, or “qubit” as used in quantum computers, and for consciousness in the Orch OR theory. **(B)** Anesthetic gases bind in non-polar regions and form their own van der Waals “dipole dispersion” forces, preventing oscillation and superposition (concept similar to Hameroff, 2020).

Figure 5 (Left) shows coupled dipoles existing as superpositions of both possible states, forming a quantum bit, or “qubit” utilized in quantum computing, and proposed to be necessary for consciousness in the Orch OR model. On the right, an anesthetic molecule (“An”) blocks the quantum dipole oscillations, preventing superposition and consciousness (Craddock et al., 2017).

Fröhlich proposed that induced electron cloud dipoles would oscillate coherently over large distances if they were (1) arrayed in periodic lattices, (2) held in a common voltage potential, and (3) pumped by ambient heat. Fröhlich predicted such dipoles would collectively oscillate (as “optical phonons”) in terahertz, gigahertz and megahertz frequency ranges, condensing to a common mode—“Fröhlich coherence”—comparable to Bose-Einstein condensation at biological temperatures (Hameroff, 1994).

Benzene (or “phenyl”) pi resonance organic rings are found in nearly all biomolecules, including lipids, proteins and nucleic acids which are generally “amphipathic” with a non-polar organic ring (either benzene/phenyl, or the more complex indole ring) at one end of the molecule, and a polar, water-soluble charge group (e.g., OH hydroxyl) on the other. Aromatic amino acids and most neurotransmitters and psychoactive molecules are amphipathic, having non-polar benzene-like (or indole-like) rings on one end of the molecule, and polar, charged groups on the other. The non-polar, “oil-like” rings have inducible dipoles which attract and coalesce, avoiding the polar, aqueous environment (“oil and water don’t mix”). Thus, proteins including tubulin have non-polar, “hydrophobic” (water-aversive) interiors with aromatic rings and other non-polar amino acids, and charged outer surfaces soluble in water. Protein inner regions including aromatic rings can support quantum effects, and are shielded from the polar, aqueous environment. Geometric arrays like microtubules can convert ambient heat to coherent oscillations in quantum-friendly aromatic pathways, proposed to extend tubulin-to-tubulin through lattice pathways, reaching mesoscopic and macroscopic scales (the “quantum underground,” Craddock et al., 2016).

**FIGURE 6:**
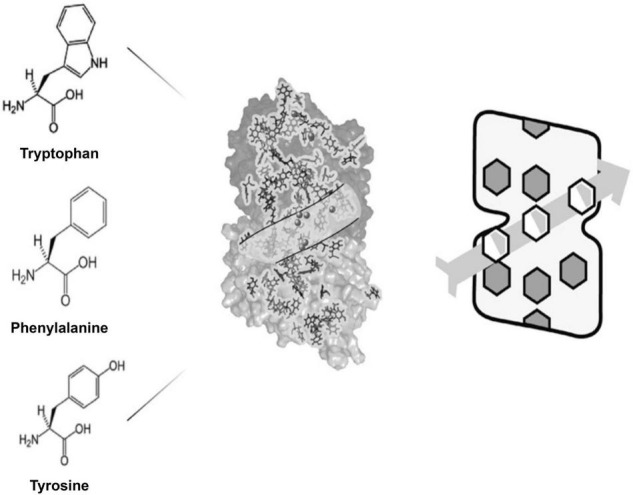
**Left:** 3 aromatic amino acids with pi resonance indole (tryptophan), and phenyl/benzene rings (phenylalanine, tyrosine). **Middle:** tubulin with its 86 pi resonance rings (spheres show anesthetic binding sites). **Right:** A simplified model representing dipole couplings among 3 rings along a helical pathway in a microtubule.

In Figure 6 on left are aromatic amino acids tryptophan, phenylalanine and tyrosine with their organic rings. In the middle is the atomic structure of tubulin with its 86 aromatic amino acids. A path through the tubulin is shown which contains binding sites for anesthetic gas molecules (spheres). On the right is a simplified version of tubulin with rings aligned along a particular winding pathway in a microtubule lattice, a pathway which could serve in quantum computing.

In quantum computing, information can exist not only as “bits” of either 1 or 0, but also as quantum superpositions of both states, quantum bits, or “qubits” of both 1 *and* 0. Multiple qubits can entangle and evolve collectively as a wavefunction until reduction/collapse to classical, definite states occurs as the solution. Orch OR is a proposed form of biological quantum computing in which collapse occurs by Penrose OR, producing “orchestrated” OR conscious moments at time t = ħ/E_G._ What are the superpositions? What is the qubit in Orch OR?

**FIGURE 7:**
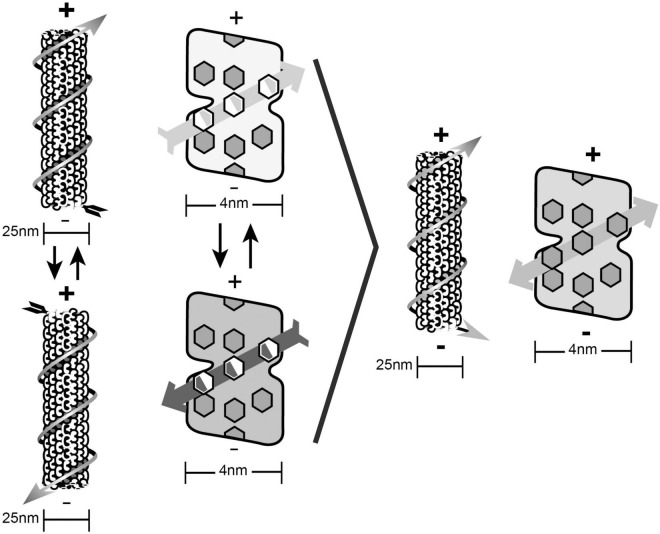
The Orch OR qubit. Helical quantum dipoles in microtubules form coherent states of one orientation or another by Fröhlich “giant dipole” oscillations. Right: Superposition of both dipole orientations form the Orch OR qubit. Separate alternate states are shown on left, superposition of both states on right. The helical pathway shown is the “5-start” pathway in the A-lattice microtubules with Fibonacci geometry (3, 5, and 8-start helical pathways).

The atomic structure of tubulin (Nogales et al., 1999) revealed 86 aromatic rings of tryptophan, phenylalanine and tyrosine, clustered densely enough to allow van der Waals quantum dipole couplings and oscillations within each tubulin, and between tubulins along pathways in microtubule lattices. Hameroff and Penrose (2014a,b) proposed the qubit model shown in Figure 7 in which Fröhlich “giant dipole” oscillations extend between adjacent tubulins along a helical path in an A-lattice microtubule. The two alternate dipole orientations, and the superposition of both orientations constitute the Orch OR qubit whose reduction may be quantified by *t* = *h*/*E*_*G*_. Helical pathway qubits resemble topological qubits which are stable and error self-correcting.

**FIGURE 8:**
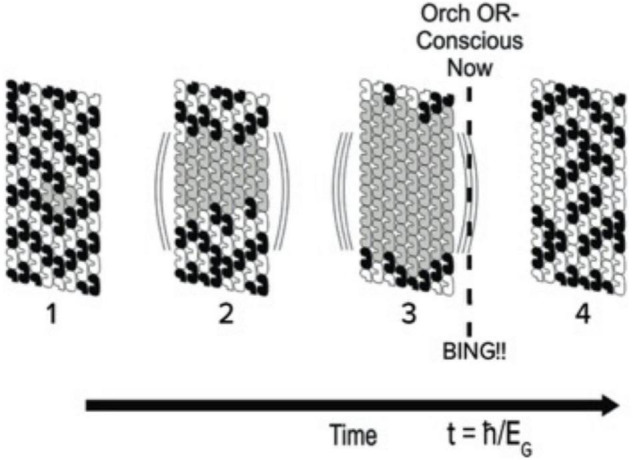
Portion of microtubule lattice in which tubulins within helical pathways may be in one of 2 classical states (black or white) with growing number of superposition states (gray). One portion of one microtubule is shown, but many microtubules may be involved in brain neurons by entanglement. At time t = ħ/E_G_, threshold is met and an Orch OR conscious moment (NOW) occurs, BING denotes phenomenal experience, or qualia.

**FIGURE 9:**
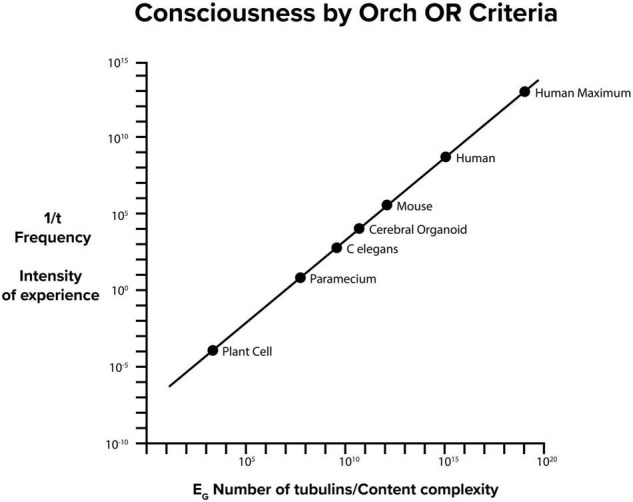
Consciousness by Orch OR criteria E_G_ = ħ/t. On the Y axis, 1/t frequency in Hz of Orch OR events also indicates intensity of experience, On the X axis, E_G_ and number of tubulins also indicate information content capacity of conscious moments.

Hameroff and Penrose (1996a) determined E_G_ for one tubulin, and used that value to calculate how many superpositioned tubulins would be required for Orch OR at any given time *t* (Figure 8). The human brain contains about 10^19^–10^20^ tubulins. If these were all entangled in one superposition E_G_, time *t* = *h*/*E*_*G*_ for Orch OR would be reached in about 10^–11^ s, a frequency of about 100 gigahertz (10^11^ Hz). This could be considered maximal possible human consciousness involving 100% of brain capacity (Figure 9), but is extremely unlikely for several reasons. What may be estimated as “ordinary” consciousness, at say 10 megahertz (10^7^ Hz) would require about 10^16^ tubulins, roughly one thousandth, 10^–3^ of brain capacity. The 10 megahertz Orch OR events are proposed to interfere to give slower events as “beat frequencies” in time frames of EEG and cognitive events, e.g., tens to hundreds of milliseconds. Indeed Orch OR can occur along a spectrum of conscious events occurring in the same brain region, and/or others by entanglement, with mixed and resonant frequencies given by *1/t* = *E*_*G*_/*ħ.*

Orch OR conscious events at higher frequency (greater *E_*G*_*) would correspond with greater experiential intensity, and also higher information capacity. Figure 9 shows E_G_, number of brain tubulins and potential frequency and intensity of experience for various organisms. In principle, Orch OR could occur at various levels of a scale-invariant hierarchy (Figures 1, 2), perhaps similar to how musical notes can occur, resonate and interfere across various scales and frequencies to produce music.

## Consciousness in the Brain

Where in the brain might Orch OR consciousness occur? The precise mechanisms underlying consciousness are perhaps most likely to reveal themselves through deviation from Hodgkin-Huxley behavior. The most likely site for that is the apex of the “perception-action” cycle in psychology, Layer V cortical pyramidal neurons.

**FIGURE 10:**
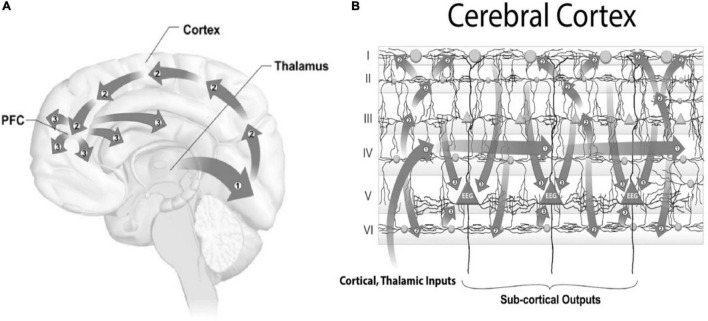
**(A)** Conscious perception occurs in three waves from thalamus, for vision (Wave 1) to V1 in the back of the brain, (Wave 2) is feed-forward to front of brain, and (Wave 3) global broadcast from front of brain which correlates with consciousness after several hundred milliseconds. **(B)** Three waves in cortex. Inputs to 6-layered cortex go (Wave 1) to layer IV, then (Wave 2) to layers I, II, III and VI, and finally (Wave 3) converging on layer V giant pyramidal neurons.

Sensory inputs (other than smell) are conveyed through thalamus, and then in 3 waves to cortex (Figure 10A). For vision, inputs from the eyes to thalamus are then relayed (Wave 1) to Visual area 1 (“V1”) in the back of the brain, and then (Wave 2) feedback through associative cortex to the front of brain, and (Wave 3) global broadcast from frontal and pre-frontal cortex. It is Wave 3 activity which appears to correlate with consciousness, although Wave 3 occurs several hundred milliseconds after sensory impingement. Because we often respond to sensory inputs within 100 milliseconds, seemingly consciously, the mainstream view in neuroscience and philosophy is that we do so non-consciously, with a subsequent false sense of conscious control. Accordingly, consciousness is considered epiphenomenal and illusory (Dennett, 1991; Wegner, 2002).

Input activity arriving in cerebral cortex (Figure 10B) is also conveyed in 3 waves to the 6 cortical layers, beginning with Layer IV (Wave 1), then to Layers I, II, III and VI (Wave 2), and finally to Layer V giant pyramidal neurons (Wave 3).

**FIGURE 11:**
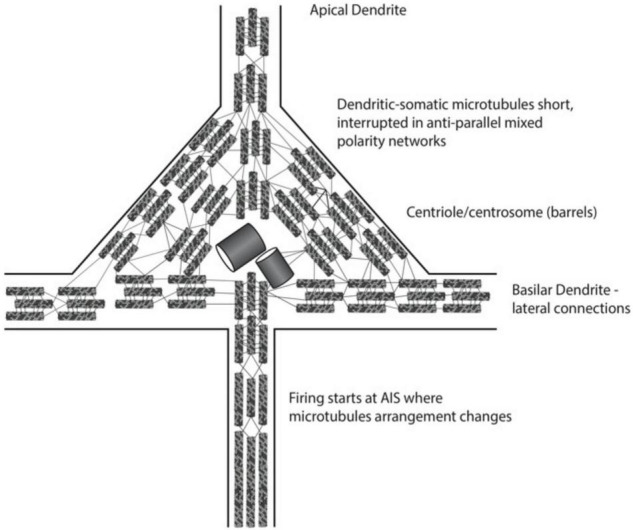
Schematic of portion of layer V giant pyramidal neuron, with cone-shaped cell body. An apical dendrite arises to the cortical surface and is a source of electroencephalography (EEG). Basilar dendrites extend laterally to other pyramidal dendrites (not seen). A single axon extends downward, conveying action potential firings, or spikes which can exert volitional, causal action.

Cortical Layer V “giant” pyramidal neurons appear to be the most likely sites for consciousness in the brain (Figure 11). They are the convergence of external and internal integration, the “apex” of the perception-action cycle, and thus a strategic location for conscious perception and action. Axonal firing outputs from Layer V pyramidal neurons descend directly to spinal cord motor “pyramidal” tracts, able to directly exert causal action in behavior. Apical dendrites arise from the tops of pyramidal cell bodies, ascend vertically to the cortical surface and collectively produce local field potentials which are detected at the brain surface or scalp as electro-encephalogram (“EEG”). Basilar dendrites extend laterally from pyramidal cell bodies/soma, forming dendritic-dendritic interactions with other Layer V pyramidal neuron basilar dendrites, and thus Layer V forms a horizontal “brain-wide web” over the entire cerebral cortex. Karl Pribram (1971) suggested this basilar dendritic-dendritic 2-dimensional web of pyramidal neurons covering the brain gave rise to coherent excitations, interference and 3-dimensional holograms relevant to consciousness. Finally, cortical Layer V pyramidal cell bodies/soma and dendrites have the largest collection of mixed polarity microtubule networks, the possible key to interference and scale-invariant processes involved in consciousness.

Microtubules in neuronal dendrites and soma are uniquely arranged, quite differently than in axons, or in all other non-neuronal biological cells. Each tubulin is a peanut-shaped dimer with alpha and beta monomers, with negative charge at alpha ends, and positive charges on beta ends. Tubulins align alpha to beta in vertical protofilaments which form microtubules, so tubulins, protofilaments and microtubules all have a net polarity—an “alpha minus” end and a “beta plus” end (Figure 7).

Microtubules are generally aligned in the same polarity, either radially, or in parallel unipolar bundles, e.g., in axons. And microtubules are generally continuous, uninterrupted. However, uniquely in all biology, microtubules in neuronal dendrites and soma are interrupted and short, and arrayed in anti-parallel networks of mixed polarity. The reason for this mixed polarity arrangement exclusively in dendritic-somatic “integration” regions of neurons is unknown. One possibility (Hameroff and Penrose, 2014a) is that anti-parallel microtubules of opposing polarities relative to the ambient neuronal membrane field will oscillate at slightly different frequencies, and generate slower interference “beats,” with effects across multiple scales, including cognitive events and EEG in tens to hundreds of milliseconds.

The transition from dendritic-somatic integration to axonal firing occurs at the “axon initiation segment” (“AIS”), a region on the proximal axon, a short distance from the soma/cell body. Membrane function changes from “analog” integration to “all-or-none” digital axonal firing, the different membrane functions apparently determined or correlated with microtubule arrangement. Proximal to the AIS, the microtubules in the axon are mixed polarity and interrupted, while distal to the AIS they are unipolar and continuous (Figure 11).

Naundorf et al. (2006) recorded from electrodes in cortical Layer V pyramidal neurons of awake, presumably conscious cats. They found variability in firing threshold on a spike-to-spike basis. The variability was attributed to noise, but there was no such deviation in control neurons. Yaron-Jakoubovitch et al. (2008) placed two electrodes in Layer V cortical pyramidal neurons *in vitro*, one in the soma, and one near the tip of the apical dendrites, 50 micrometers away. They recorded electromagnetic “noise” that was highly correlated between the two sites, suggesting a common, coherent source, e.g., stemming from the cytoskeleton as in the Singh et al. (2021) study. Thus “bottom-up” cytoskeletal activities are not noise, and may exert conscious causal modulation on higher level, otherwise non-conscious activities.

**FIGURE 12:**
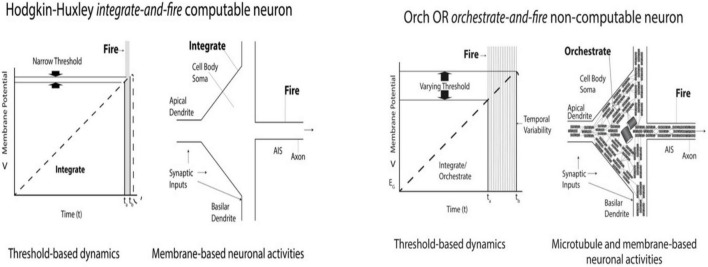
**Left:** Diagram and schematic of Hodgkin-Huxley integrate-and-fire neuronal activity. Narrow threshold and temporal firing windows reflect algorithmic “computable” behavior. There is no deviation from algorithmic, Hodgkin-Huxley behavior. Next, a schematic pyramidal neuron (apex pointing left, axon to right) with only its surface membrane for algorithmic Hodgkin-Huxley behavior. **Right:** Recordings from cortical pyramidal neurons in awake cats show widely variable membrane potential thresholds and time windows which deviate from Hodgkin-Huxley behavior (Naundorf et al., 2006), and are thus “non-computable.” Next: Schematic of pyramidal “orchestrate-and-fire” neuron with interior mixed polarity microtubule networks, suggested in Orch OR to mediate consciousness and cause non-computable deviation from Hodgkin-Huxley behavior through variability in axonal firing threshold (interpreted from Naundorf et al., 2006).

Figure 12 (Left) shows a diagram of an algorithmic Hodgkin-Huxley “integrate-and-fire” neuronal event, with a very narrow membrane potential threshold, and temporal firing window. There is no deviation from algorithmic, Hodgkin-Huxley behavior. Also on the left is a schematic of a Layer V pyramidal neuron (rotated sideways/counter-clockwise, compared to Figures 10B, 11). Only the surface membrane, upon which Hodgkin-Huxley is exclusively based, is shown.

Figure 12 (Right) shows a diagram of a non-computable (non-algorithmic) “orchestrate-and-fire” neuron which deviates from Hodgkin-Huxley behavior, showing variability in membrane threshold potential and temporal firing as found by Naundorf et al. (2006) in Layer V pyramidal neurons of awake cats. On the Right, a schematic of a Layer V pyramidal neuron shows internal networks of mixed polarity microtubules, proposed in Orch OR to host conscious processes which causally modulate axonal firing, deviating from Hodgkin-Huxley behavior.

As shown by Naundorf et al. (2006), a “non-computable” factor causally modulates Hodgkin-Huxley behavior of the integrated membrane potential of Layer V pyramidal neurons of awake cats to regulate axonal firing. Is the non-computable effect related to consciousness? Is it high frequency oscillations in dendritic-somatic microtubules? Does anesthesia (which blocks consciousness while sparing non-conscious brain activities) reverse deviation from Hodgkin-Huxley behavior, reverting to non-conscious algorithmic “auto-pilot” behavior? Does consciousness arise from quantum processes in dendritic-somatic microtubules in a brain-wide web of Layer V pyramidal neurons? Consideration of these possibilities may lead to a new paradigm in neuroscience.

## Discussion – The Need for a New Paradigm in Neuroscience

For almost 70 years the brain has been viewed as a complex computer of simple neurons, specifically of Hodgkin-Huxley “integrate-and-fire” threshold logic devices, with information processing mediated exclusively by membrane and synaptic activities. This mainstream view ignores neuronal interiors, and any aspect of biology, treating neurons as essentially inanimate objects. Non-living electronic circuits and networks comprised of threshold logic devices (“artificial neural networks”) can indeed “compute” and in principle account for some cognitive brain functions. But the “brain-as-computer” notion has severe limitations and paradoxes compared to human and animal brain functions. It is suggested here that these limitations and paradoxes may be resolved by a new paradigm, a scale-invariant hierarchy which includes deeper level, faster and smaller intra-neuronal quantum and classical processing in microtubules and other cytoskeletal components *inside brain neurons*. This deeper level processing involves unitary quantum states which are quintessentially biological, close to the fundamental nature of both life and consciousness.

Here we review the limitations and paradoxes of the brain-as-computer approach, and how they can be resolved in the new paradigm.

### Fragmented and Unbound Consciousness

The brain-as-computer approach fails to account for binding and unity of conscious experience (von der Malsburg, 1999; Mashour, 2013). For example, various aspects of visual inputs (e.g., shape, color, motion and meaning) are processed in different regions of cortex, and at different times, yet we consciously perceive integrated, spatiotemporally unified visual objects and scenes. The sense of “self” is consciously perceived as a unified coherent oneness, despite the lack of any actual unity among recognized neuronal activities.

#### New Paradigm Explanation

Consciousness, cognitive binding and the sense of self involve quantum processes unified through spatiotemporal coherence, condensation and entanglement. For example, if one component in a Bose Einstein condensate is perturbed, all other components are perturbed—as if the condensate “feels it.” Quantum dipole, spin and optical processes can operate at warm temperatures in non-polar, hydrophobic (water-aversive) “quantum channels” in protein interiors, for example comprised of aromatic amino acid rings of delocalized pi electron resonance clouds and other non-polar groups (the “quantum underground”). In microtubules, quantum channels within each tubulin can link to those in neighboring tubulins in the microtubule lattice, providing mesoscopic and macroscopic quantum channel pathways (e.g., Figure 7).

### Memory

In the brain-as-computer approach, memory is ascribed to synaptic plasticity regulating flow of electrochemical information through networks of neurons. However, membrane proteins involved in synaptic sensitivity are transient, re-cycled over hours to days, and yet memories can last lifetimes. We also know that memory is somehow distributed throughout the cortex and may be linked to other memories by the hippocampus. How memory is encoded and recalled into consciousness are unknown.

#### New Paradigm Explanation

It would be efficient and convenient if consciousness occurred in the same medium and location where memory is encoded. Could memory be stored in microtubule lattices? It turns out that dendritic-somatic microtubules have an enormous capacity for encoding information from synaptic and other “top-down” inputs, can be extremely stable over lifetimes, encode synaptic inputs, e.g., the “tubulin code” (Janke, 2014) and are likely sites for consciousness. Memory capacity: Figure 3 shows microtubules composed of tubulin proteins which are each in one of two states, black or white. With ∼10^8^ tubulins per neuron, there are 2 × 10^8^ possible tubulin binary states per neuron. These states oscillate and change, and wouldn’t be useful in memory, but they could oscillate and change on a background of memory encoded in each tubulin. In a brain microtubule each tubulin could have 17 different possible genetic isoforms (Lee et al., 1986), and may also undergo 4 types of post-translational modifications, as well as phosphorylation and other modifications (the “tubulin code,” see above). Thus each microtubule may be a heterogeneous mosaic and thus able to encode vast amounts of information, each tubulin encoding one of at least ∼10^22^ possible states. With ∼10^8^ tubulins per neuron, memory capacity in a single neuron is vast (∼10^8^ raised to the power of 10^22^). Stability of memory in neuronal microtubules: Microtubules in cells other than neurons have repetitive cycles of mitotic cell division in which structural microtubules disassemble, and then re-assemble as mitotic spindles which guide chromosomes. Spindle microtubules then disassemble and re-assemble to resume their structural role. Mosaic lattice relationships of tubulins within microtubules in dividing cells are thus repeatedly “scrambled,” so memory would be lost. However, neurons don’t divide, and neuronal microtubules do not undergo cycles of disassembly and reassembly. Further, dendritic-somatic microtubules are “capped” which prevents “treadmilling,” assembly at one end, and disassembly at the other. Thus mosaic lattice arrangements of particular tubulins in dendritic-somatic microtubules are preserved over time, potentially over the life of the organism. Synaptic input encoding: Synaptic transmissions trigger post-synaptic events including activation of the hexagonal holoenzyme calcium-calmodulin kinase II (“CaMKII”) which can contact and phosphorylate up to 6 tubulins in a microtubule lattice simultaneously and individually, i.e., up to 6 bits of information. Thousands of CaMKII molecules are activated with each synaptic event in a neuron, so microtubules are a viable candidate for distributed memory storage (Craddock et al., 2012a,b). Phosphorylation can impart nuclear spin which is stable and proposed to store memory (Fisher, 2015), and could impart memory in specific tubulins by CaMKII (Craddock et al., 2012a,b).

### Consciousness as Axonal “Firings”?

Neuronal dendrites and cell bodies/soma receive and integrate synaptic inputs as membrane potentials to a threshold for axonal “firings,” or “spikes.” Likening the brain to digital computers forces the assumption that axonal “firings” are the digital currency of cognition and consciousness. But integration (or “orchestration”) in dendrites and soma of neurons (which regulate and *cause* axonal firings) correlates better with consciousness than do firings. Electro-encephalography (“EEG”) including gamma synchrony, a marker of consciousness, is generated primarily from local field potentials of dendrites, specifically apical dendrites of Layer V giant pyramidal cell neurons. General anesthetics which selectively block consciousness act on dendritic receptors and don’t affect the firing process.

#### New Paradigm Explanation

Consciousness is most likely to occur in a scale-invariant hierarchy originating in dendritic-somatic networks of mixed-polarity microtubules in Layer V cortical pyramidal neurons. Quantum dipole oscillations in aromatic “organic” rings inside tubulin can extend through microtubule pathways and be orchestrated, entangled and synchronized until reaching threshold for quantum state (“objective”) reductions and conscious moments. EEG is predicted to derive from interference “beats” of higher frequency vibrations in microtubules (Hameroff and Penrose, 2014a). Consciousness seems more likely to occur in a deeper, faster level in dendrites and soma of Layer V pyramidal neurons, with firings conveying the results of consciousness to the next layer of synapses and effector organs.

### Epiphenomenal and Illusory

Consciousness apparently occurs “too late” for real-time conscious action. Brain activity correlating with sensory inputs occurs 300–500 milliseconds after the inputs, and yet we often respond to those inputs, seemingly “consciously,” at around 100 milliseconds. Accordingly, the conventional view in science and philosophy is that we respond non-consciously, with a false illusion of conscious control (Dennett, 1991; Wegner, 2002). Free will for real time events is impossible.

#### New Paradigm Explanation

Research on awake neurosurgical patients with their brains exposed (Libet et al., 1979) showed that real time conscious perception at the time of a sensory evoked potential 30 milliseconds after sensory impingement requires 300–500 milliseconds of ongoing cortical activity, what Libet termed “neuronal adequacy.” The brain somehow “knows” at 30 milliseconds whether or not activity will persist to “neuronal adequacy” at 300–500 milliseconds. Libet concluded information was referred “backward in time” from neuronal adequacy to the earlier evoked potential, enabling conscious perception and action within 100 milliseconds, allowing the possibility of free will. Mainstream neuroscience rejected Libet’s results, largely because the concept of backward time effects seemed impossible. But quantum physics allows for temporal non-locality, and the very nature of the flow of time may depend on consciousness, which may in turn depend on sequences of quantum state reductions as suggested in Orch OR. In providing an account of his theory of quantum state “objective reduction,” Roger Penrose (1989, 2022) deduced a retroactive, “backward in time” referral which can account for such effects, offering a scientific basis for real-time conscious control and free will (Hameroff, 2012).

### Computable, Algorithmic and Predictable

The brain-as-computer of simple Hodgkin-Huxley neurons is algorithmic, “robot-like,” seeming to preclude insight, creativity and free will, as well as consciousness.

#### New Paradigm Explanation

Deviation from Hodgkin-Huxley behavior may be a marker, or correlate of consciousness. Naundorf et al. (2006) showed deviation from such algorithmic behavior in cortical layer V pyramidal neurons of awake, presumably conscious cats (Figures 9–11). The standard explanation for the deviation is intra-neuronal “noise,” however, control neurons in culture had no such deviation. Singh et al. (2021) showed functional effects of intra-neuronal electromagnetic oscillations in megahertz and kilohertz frequencies. Yaron-Jakoubovitch et al. (2008) measured intra-neuronal electromagnetic fields with electrodes at two locations inside cortical Layer V pyramidal neurons, one in the soma/cell body, and the other near the tip of the apical dendrite, 50 micrometers away. The electromagnetic “noise” activity in the two locations was highly correlated, perhaps because it was due to collective cytoskeletal oscillations, as Singh et al. (2021) had shown could regulate membrane and synaptic effects. A deeper level of hidden circuits appears to play an important role in neuronal function, modulating Hodgkin-Huxley behavior, particularly in cortical Layer V pyramidal neurons. This is where consciousness seems most likely to occur, observable as deviation from Hodgkin-Huxley behavior.

### The “Hard Problem”

The essential quality of consciousness is phenomenal experience, including feelings and awareness, comprised of what philosophers term “qualia.” Could the world have been populated by non-conscious “zombies” with no feelings nor inner life? If so, what is the essential difference between their brains, and those of ours with full, rich conscious experience? Chalmers (1996) described the mysterious nature of conscious experience as the “hard problem,” in contrast to so-called “easy problems” like sensory discrimination, information integration, purposeful movement, focusing attention and others which may be, at times at least, entirely non-conscious, “autopilot” functions. The Hodgkin-Huxley brain-as-computer approach can in principle account for “easy problems,” non-conscious autopilot cognition, but not the hard problem of conscious experience.

#### New Paradigm Explanation

Zombies would have Hodgkin-Huxley membrane and synaptic-neuronal activities, but not Orch OR or other deeper, faster quantum activities in the cytoskeleton. Atoms and small particles appear to exist as quantum superpositions of multiple possibilities, but yet we observe discrete, definite objects in our perceived “classical” world. Some take this to imply that measurement, or conscious observation causes “quantum state reduction,” that consciousness “collapses the wavefunction,” a dualist view that places consciousness outside science.

Neuroscientists and philosophers have resorted to panpsychism to address the hard problem, the notion that qualia are somehow fundamental, e.g., that atoms and particles have qualia (e.g., Koch, 2012). Roger Penrose (1989) had suggested the answer was even more fundamental, that quantum state reduction (which selects particular states of atoms and particles) occurs spontaneously, and results in (proto-) conscious moments, or qualia. In Orch OR these are combined, unified and “orchestrated” in brain microtubules to give full, rich conscious moment. Quantum processes in microtubules would upwardly influence axonal firings, and larger and slower processes in the brain’s scale-invariant hierarchy. They would also extend downward to the most fundamental level of the universe, spacetime geometry. Inclusion of Orch OR in dendritic-somatic microtubules in Layer V pyramidal cells could address the “hard problem.”

## Conclusion

Mainstream approaches in neuroscience and philosophy view the brain as a complex computer of simple Hodgkin-Huxley “integrate-and-fire” neurons. But these approaches fail to account for consciousness, important aspects of cognition, nor intelligent behaviors of single cell organisms which lack synaptic connections, but use their cytoskeleton to sense and act. Considering only neuronal membrane, synaptic activities and axonal firings as “bits,” the brain-as-computer is an insult to neurons, to life itself (which may derive from quantum coherence in organic chemistry, e.g., Schrödinger, 1944; Hameroff, 2017), and to consciousness, quite possibly a fundamental feature of the universe (Penrose, 1989; Koch, 2012). A new paradigm is needed in neuroscience, e.g., one which depends on the neuronal cytoskeleton in a scale-invariant hierarchy, extending some 15 orders of magnitude downward in size into quantum vibrations in cytoskeletal microtubules, and further downward to the physics of the universe (Figures 1, 2).

The key neuroscientific features of the proposed new paradigm are quantum and classical information processing in microtubules inside brain neurons, most specifically and most likely in dendritic-somatic mixed polarity networks of microtubules in cortical Layer V pyramidal neurons. These neurons’ cell bodies and basilar dendrites are part of a 2-dimensional grid extending throughout the cortex, covering the brain, a nexus of sensory and internal stimuli, generating EEG and controlling motor outputs and behavior. Layer V pyramidal neuron cell bodies contain the largest known networks of mixed polarity microtubules, thought optimal for “quantum interference beats,” and fail to follow Hodgkin-Huxley behavior, the failure a possible tell-tale sign of consciousness.

Recordings from cortical Layer V pyramidal neurons in awake, presumably conscious animals show significant deviation from neuronal computational behavior, i.e., from algorithmic Hodgkin-Huxley “integrate-and-fire” responses (Naundorf et al., 2006). Some additional factor appears to causally modulate algorithmic “auto-pilot” behavior. The Orch OR theory suggests it is consciousness due to “orchestrated objective reduction” in dendritic-somatic microtubules, e.g., in Layer V pyramidal neurons that does so. An “orchestrate-and-fire” model of pyramidal neuron activity is presented in which Orch OR quantum processes in microtubules *causally modulate* triggering of axonal firings, or “spikes,” a “non-computable” effect ideally located for consciousness to regulate behavior.

Mixed polarity microtubule networks offer the capability for interference among microtubules oscillating at slightly different frequencies due to opposite orientations in the surrounding ambient electromagnetic field. This interference may generate slower, self-similar “beat frequencies” at larger scales relevant to cognitive events and EEG—a scale-invariant hierarchy (Hameroff and Penrose, 2014a). The brain may be more like a self-organizing hologram, or orchestra than algorithmic computer.

The origin of consciousness, and of life, appear to derive from quantum dipole oscillations and optical effects among organic rings buried inside proteins and other biomolecules, shielded from polar charges, providing “quantum channels” (or “quantum underground,” Craddock et al., 2016) pervading living systems. As suggested by Schrödinger (1944) and Fröhlich (1968, 1970, 1975) quantum dipole oscillations arrayed in geometric lattices can convert ambient heat to synchronized vibrations, resonate and oscillate in terahertz, gigahertz, megahertz and slower frequencies, a scale-invariant hierarchy in which Orch OR consciousness can occur.

Finally, the new paradigm points to medical applications by addressing microtubule and cytoskeletal origins of neuropathology including Alzheimer’s disease, traumatic brain injury and depression. In Alzheimer’s, cognitive dysfunction correlates with loss of the microtubule-associated protein “tau” from microtubules, destabilizing them and causing their depolymerization. In traumatic brain injury, microtubules, particularly those in long axons are often fractured. And antidepressants like fluoxitene (“Prozac”) take weeks to take effect as the dendritic-somatic cytoskeleton structurally reorganizes (Bianchi et al., 2009). Indeed most types of psychiatric illnesses appear to have abnormalities of the cytoskeleton (Marchisella et al., 2016). Therapies should be aimed at the cytoskeleton, for example low intensity transcranial ultrasound (megahertz mechanical vibrations) applied to the brain has been used to safely treat various mental and cognitive disorders. The mechanism is unclear, but may involve resonating megahertz vibrations in microtubules (Sanguinetti et al., 2020).

Mainstream views of membrane receptors and ion channels mediating cognition and consciousness are quite literally shallow and superficial. Neuroscience needs a new paradigm for brain function, one which includes quantum and classical information processing in the neuronal cytoskeleton.

## Data Availability Statement

The original contributions presented in the study are included in the article/supplementary material, further inquiries can be directed to the corresponding author.

## Author Contributions

The author confirms being the sole contributor of this work and has approved it for publication.

## Conflict of Interest

The author declares that the research was conducted in the absence of any commercial or financial relationships that could be construed as a potential conflict of interest.

## Publisher’s Note

All claims expressed in this article are solely those of the authors and do not necessarily represent those of their affiliated organizations, or those of the publisher, the editors and the reviewers. Any product that may be evaluated in this article, or claim that may be made by its manufacturer, is not guaranteed or endorsed by the publisher.
